# Significant association between insertion/deletion polymorphism of the angiotensin-convertig enzyme gene and ankylosing spondylitis

**Published:** 2012-07-26

**Authors:** Ahmet Inanır, Serbulent Yigit, Sengul Tural, Sibel Demir Ozturk, Songul Akkanet, Abdulkadir Habiboğlu

**Affiliations:** 1 Gaziosmanpaşa University, Faculty of Medicine, Dept. of Physical Medicine and Rehabilitation, Tokat, Turkey; 2 Gaziosmanpaşa University, Faculty of Medicine, Department of Medical Biology, Tokat, Turkey; 3 Ondokuz Mayis University, Faculty of Medicine, Department of Medical Biology, Section of Medical Genetics, Samsun, Turkey

## Abstract

**Purpose:**

Ankylosing spondylitis (AS) is a chronic inflammatory rheumatic disease that characteristically affects the sacroiliac joints and the spine. Also iritis and uveitis can be serious complications of AS that can damage the eye and impair vision. The exact pathogenesis of AS remains poorly understood but genetic factors play a key role in its development. Human leukocyte antigen B27 (*HLA-B27*) is the major genetic susceptibility marker in AS. To our knowledge, angiotensin converting enzyme (*ACE*) gene I/D polymorphisms have not yet been investigated in AS patients in Turkish population.This study was conducted in Turkish patients with AS to determine the frequency of I/D polymorphism genotypes of angiotensin converting enzyme gene.

**Methods:**

Genomic DNA obtained from 262 persons (122 patients with ankylosing spondylitis and 140 healthy controls) was used in the study. *ACE* I/D polymorphism genotypes were determined by using polymerase chain reaction with specific primers.

**Results:**

There was statistically significant difference between the groups with respect to genotype distribution (p<0.001). When we examine *ACE* genotype frequencies according to the clinical characteristics there was a statistically significant association between DD genotype and ocular involvement (p=0.04) also sacroiliac joint involvement (p=0.03).

**Conclusions:**

As a result of our study, angiotensin converting enzyme gene I/D polymorphism DD genotype could be a genetic marker in ankylosing spondylitis in a Turkish study population.

## Introduction

Ankylosing spondylitis (AS) is the prototypical form of seronegative spondylarthropaties, a group of disorders that involves chronic inflammation of the sacroiliac joints and spine leading to progressive stiffening of the spine and ankylosis [1]. The most frequent extraarticular manifestation in spondyloarthropathies is eye involvement, which is found in 30%–50% of patients [2]. Acute anterior uveitis is the most common extraspinal lesion of AS occurring in the course of the disease. Uveitis can lead to serious complications that can compromise the visual function [1]. Prevalence of uveitis increases with duration of disease. Typical eye involvement is sudden-onset unilateral anterior uveitis (iridocyclitis) [1]. Acute anterior uveitis may occur as a minimal variation or initial symptom of spondyloarthropathies. Human leukocyte antigen B27 (HLAB 27) positive patients in particular -mostly those in the subgroup with ankylosing spondylitis- are affected. Posterior uveitis (choroiditis or retinitis) is commonly associated with infection, sarcoidosis, and Behçet’s disease [2,3]. In Turkey, the prevalences of AS and related spondyloarthritis have been determined as 0.49% and 1.05% [4].

Angiotensin converting enzyme (ACE; also known as peptidyl dipeptidase A or kininase II), encoded by the *ACE* gene (GenBank NM_000789.2). *ACE* is located on the long arm of chromosome 17 and can be expressed in multiple tissues [5]. *ACE* contains a polymorphism based on the presence (insertion, I) or absence (deletion, D) within intron 16, of a 287 base pair ALU repeat sequence; resulting in 3 genotypes: DD and II homozygous and ID heterozygous. Plasma ACE levels vary with polymorphism; individuals homozygous for the D allele have the highest levels of enzyme, those homozygous for the insertion allele ve the lowest and heterozygous subjects have an intermediate level [6-10]. The local renin-angiotensin system (RAS) in the vessel walls plays a crucial role in the endothelial control of vascular tonus and, contributes to the inflammatory process via stimulation of cytokine production [11]. ACE is a regulatory component of the RAS by hydrolysing inactive angiotensin I to the active angiotensin II and inactivating the bradykinin [12]. ACE has been identified as a membrane-bound enzyme in several types of cells. It is also present in a circulating form, produced by vascular endothelial cells, in biologic fluids such as plasma. The levels of tissue and circulating ACE activities are under the genetic control [13]. This polymorphism affects circulating and tissue levels of ACE. The changes in ACE activity could affect endothelial function also (I/D) polymorphism of the *ACE* gene determines the plasma and tissue levels of ACE especially in the synovial fluid. Based on these findings we aimed that the genotype of *ACE* in AS patients may be a determining factor in pathogenesis. To our knowledge, *ACE* I/D polymorphisms have not yet been investigated in AS patients in Turkish population

## Methods

### Study population

This study included 122 AS patients and 140 controls recruited from the department of Physical Medicine and Rehabilitation, Gazi Osmanpaşa University in Tokat, Turkey. Informed consent in accordance with the study protocol was approved by the ethics committee of Gazi Osmanpaşa University Medical Faculty. Diagnosis of AS was based on the modified New York criteria [13]. All patients signed a written consent form after being informed about the details of the study. A complete clinical evaluation was done for all patients. The controls were selected by excluding the diagnosis of AS. All the individuals in the control group were healthy. Data collection sheet included information such as age, disease duration,smoking status, exercise habit and several clinical characteristics. Turkish version of Bath Ankylosing Spondylitis Diseaes Activity Index (BASDAI) was evaluated. Individual features of patients with AS and controls were summarized in Table 1.

**Table 1:** Demographic variables and baseline characteristics of the patients.

**Individual characteristics**	**Mean±SD**	**Min-Max**
Average age of patients	38.64±9.21	19–65
Average age of controls	38.07±13.66	18–65
Diagnosis age, years	32.64±9.66	30.75–34.53
Disease duration, years	6.252±5.16	5.24–7.26
ESR, mm/h	13.569±1.33	15.65–20.96
Modified Schober, cm	3.957±1.507	3.66–4.25
Chest Expansion, cm	2.655±1.068	2.44–2.86
BASDAI	3.178±1.336	2.91–3.44

ESR: erythrocyte sedimentation rate. BASDAI:Turkish version of Bath Ankylosing Spondylitis Diseases Activity Index.

### Genotype determination

DNA was extracted from 2 ml venous blood according to kit procedure (Sigma, Steinheim, Germany) and stored at −20 °C. *ACE* genotypes were determined by polymerase chain reaction (PCR). Reactions were performed with 10 pmol of each primer: sense oligo: 5′-CTG GAGACCACT CCCATC CTT TCT-3′and antisense oligo: 5′-GAT GTG GCC ATC ACATTC GTC AGAT-3′ in a final volume of 50 μl, containing 3 mM MgCl_2_, 50 mM KCl, 10 mMTris-HCl pH 8.4, 0.1 mg/ml gelatin, 0.5 mM of each dNTP (Geneun, Ludwigshafen, Germany), and 2.5 u Taq DNA polymerase (Fermentas, Leon-Rot, Germany). DNA was amplified for 30 cycles with denaturation at 94 °C for 1 min, annealing at 60 °C for 1 min 45 s, and extension at 72 °C for 1 min 30 s using a thermal cycler (Techne, Foster City, CA). PCR products were analyzed on 2% agarose gels after staining with ethidium bromide. In the absence of the 287 bp insertion in intron 16 of *ACE*, this PCR method resulted in a 190 bp product (D allele) and in the presence of insertion, produced a 490 bp product (I allele). In heterozygous samples, 2 bands (490 and 190 bp) were detected along with a third fragment of intermediate size. To validate the accuracy and reproducibility of this method, each PCR reaction included internal controls for each genotype. Second PCR was performed to confirm samples which results are not clear. Also, to confirm the accuracy of the genotyping, repeated analysis was performed on randomly selected samples. No discrepancies were found.

### Statistical analysis

Analysis of the data was performed using the computer software SPSS 15.0 (SPSS, Chicago, IL) and OpenEpi Info software package program [14]. Continuous data were given as mean±SD (standart deviation) and (min-max). The frequencies of the alleles and genotypes in patients and controls were compared with χ^2^ analysis. Odds ratio (OR) and 95% confidence intervals (CIs) were calculated. A p-value smaller than 0.05 (two-tailed) was regarded as statistically significant. Power analysis was done by using Minitab 15.0 package program. Hardy–Weinberg equilibrium was assessed by χ^2^ analysis.

## Results

Demographic variables and baseline characteristics of patients were given in Table 1. The mean age±standard deviation (SD) was 38.64±9.21 in patients and 38.07±13.66 in control group, respectively. There were 59 (48.36%) female and 63 (51.63%) male in patient group, in the control group it was 79 (56.42%) and 61 (43.57%), respectively. In power analysis, the power of D allele comparison between patients and controls was over 90% in 95% confidence interval. ACE genotype distribution was in agreement with the Hardy–Weinberg expectations (p>0.05). Clinical characteristics of AS patients were given in Table 2.

**Table 2:** Clinical findings of AS patients.

**Clinical characteristics**	**Patients (n=122; %)**
HLAB27 positivity	91 (74.5)
Pain presence	99 (81.37)
Exercise habit	30 (24.5)
Smoking status	17 (13.7)
Bamboo spine	24 (20)
Dorsal kyphosis	25 (20.6)
Peripheral involvement	73 (59.75)
Hip involvement	45 (37.25)
Cervical involvement	18 (14.7)
Sacroiliac joint involvement	10 (7.8)
Extraarticular involvement	23 (18.60)
Cardiac involvement	5 (3.9)
Ocular involvement	18 (14.7)
Familial history	22 (17.64)
Syndesmophtes	31 (25.5)
Oral aphthae	26 (21.56)
	86 (70.58)

Table 3 presents the distribution of *ACE* I/D genotypes in patients and control groups. There was statistically significant difference between the groups with respect to genotype distribution (p<0.001). The D allele frequency was obserwed as 67.21% and the I allele was 32.79% in the patients group, it was 36.07% and 63.93%, respectively, in the control group. In addition, when a comparison was made between genotypes and the demographic subgroups of patients with AS, there was a statistically significant association between DD genotype and ocular involvement (p=0.04), also sacroiliac joint involvement (p=0.03; Table 4).When we investigateed *ACE* DD genotype frequencies versus ID+II genotype according to the clinical characteristics in AS patients there was a higher statistically significant association between DD genotype and ocular involvement (p=0.02), also sacroiliac joint involvement (p=0.01; Table 5).

**Table 3:** Disribution of *ACE* I/D polymorphism and allele frequencies between AS patients and controls.

**Genotype**	**Patients (n=122; %)**	**Controls (n=140; %)**	**χ^2^**	**p-value**	**OR (95%CI)**
DD	61 (50)	29 (20.70)		**p<0.001**	
II	19 (15.57)	68 (48.57)			
ID	42 (34.42)	43 (30.70)			
DD+ID:II	103:19	72:68	32.00	**p<0.001**	5.087 (2.845–9.365)
ID+II:DD	61:61	111:29	24.79	**p<0.001**	0.262 (0.1513–0.4495)
Allele frequency					
D	164 (67.21)	101 (36.07)	21.64	**p<0.001**	2.426 (1.665–3.549)
I	80 (32.79)	179 (63.93)			

The results that are statistically significant are typed in bold.

**Table 4:** *ACE* genotype frequencies according to the clinical characteristics in AS patients (n=122).

		***ACE* genotypes of patients (n=122)**			
**Clinical characteristics**	**Status**	**DD**	**ID**	**II**	**p-value**
HLAB27 positivity	yes	21 (17.2)	14 (11.50)	3 (2.46)	p>0.05
	no	41 (33.6)	27 (22.13)	16 (13.11)	
Exercise habit	yes	20 (16.39)	14 (11.47)	4 (3.27)	p>0.05
	no	42 (34.45)	27 (22.13)	15 (12.29)	
Bamboo spine	yes	16 (13.11)	8 (6.55)	7 (5.73)	p>0.05
	no	46 (37.74)	33 (27.04)	12 (9.83)	
Dorsal kyphosis	yes	13 (10.65)	8 (6.55)	6 (4.94)	p>0.05
	no	49 (40.16)	33 (27.05)	13 (10.65)	
Sacroiliac joint involvement	yes	46 (37.71)	35 (28.68)	17 (13.93)	**p=0.03**
	no	16 (13.11)	6 (4.94)	2 (1.63)	
Hip involvement	yes	26 (21.03)	11 (9.01)	8 (6.86)	p>0.05
	no	36 (29.50)	30 (24.59)	11 (9.01)	
Cervical involvement	yes	9 (7.37)	3 (2.45)	4 (3.29)	p>0.05
	no	53 (43.44)	38 (31.14)	15 (12.31)	
Cardiac involvement	yes	2 (1.63)	3 (2.45)	0	p>0.05
	no	60 (49.20)	38 (31.15)	19 (15.57)	
Ocular involvement	yes	14 (11.47)	3 (2.45)	1 (0.85)	**p=0.04**
	no	48 (39.34)	38 (31.14)	18 (14.75)	
Syndesmophtes	yes	14 (11.47)	9 (7.37)	9 (7.37)	p>0.05
	no	48 (39.34)	32 (26.22)	10 (8.20)	
Shaft presence	yes	11 (9.05)	9 (7.37)	6 (4.91)	p>0.05
	no	51 (41.8)	32 (26.22)	13 (10.65)	

The results that are statistically significant are typed in bold.

**Table 5:** *ACE* DD genotype frequencies versus ID+II genotype according to the clinical characteristics in AS patients (n=122).

**Clinical characteristics**	**Status**	**DD**	**ID+II**	**p-value**
HLAB27 positivity	yes	21 (17.2)	17(11.47)	p>0.05
	no	41 (33.6)	43 (22.13)	
Exercise habit	yes	20 (16.39)	18 (11.47)	p>0.05
	no	42 (34.42)	42 (22.13)	
Bamboo spine	yes	16 (13.11)	15 (6.55)	p>0.05
	no	46 (37.70)	45 (27.04)	
Dorsal kyphosis	yes	13 (10.65)	14 (6.55)	p>0.05
	no	49 (40.16)	46 (27.04)	
Sacroiliac joint involvement	yes	46 (37.70)	53 (28.68)	**p=0.02**
	no	16 (13.11)	17 (4.91)	
Hip involvement	yes	25 (20.49)	23 (12.29)	p>0.05
	no	37 (30.32)	37 (21.31)	
Cervical involvement	yes	9 (7.37)	7 (2.45)	p>0.05
	no	53 (43.44)	53 (31.14)	
Cardiac involvement	yes	2 (7.37)	3 (2.45)	p>0.05
	no	60 (49.18)	57 (31.14)	
Ocular involvement	yes	14 (11.47)	4 (2.45)	**p=0.01**
	no	48 (39.34)	56 (31.14)	
Syndesmophtes	yes	14 (11.47)	18 (7.37)	p>0.05
	no	48 (39.34)	42 (26.22)	
Shaft presence	yes	11 (9.01)	15 (7.37)	p>0.05
	no	51 (41.8)	45 (26.22)	

The results that are statistically significant are typed in bold.

## Discussion

Ankylosing spondylitis (AS) is a chronic inflammatory disease [15]. The principal musculoskeletal lesions associated with AS are sacroiliitis, synovitis and enthesithis. The ocular, intestinal, pulmonary and neurologic complications make it a multisystemic disease [16]. The prevalence of AS ranges from 0.1% to 6.0% across different populations [1]. In the Middle East, lower figures were reported from Arab countries, that is, United Arab Emirates (UAE) 0.5%, Saudi Arabia 2.6%, Kuwait 4%, Iraq 2.1%, Lebanon 1.4%, Tunisia 3.2%, and Syria 1.4% [3]. On the other hand, a remarkably higher percentage was found in Yemeni population (17%) In Turkey, the prevalences of AS and related spondyloarthritis have been determined as 0.49% and 1.05%, respectively [4].

Genetic factors play a key role in AS development. The human leukocyte antigen (HLA-B27) is a class I antigen of the major histocompatibility complex, and it is strongly associated with ankylosing spondylitis and other related spondyloarthropathies [3]. ACE is a key regulator in inflammatory signal transduction pathways. Insertion-deletion (I/D) polymorphism of *ACE* determines the plasma and tissue levels of ACE especially in the synovial fluid [17]. In this study, the distribution of *ACE* I/D polymorphism genotypes were analyzed in AS patients in a Turkish population to assess its possible role in the pathogenesis of AS. To our knowledge, this is the first study to evaluate the prevalence of *ACE* among AS patients living in Turkey. The present study indicates that the percentage of *ACE* I/D polymorphism allele and the distribution of genotypes are significantly different between patients and controls. Clinical findings of our patients were accordance with the literature. Also we found statistically significant association between DD genotype and ocular involvement (p=0.04) and also sacroiliac joint involvement (p=0.03). Generally in patients with AS the prevalence of uveitis is approximately 30% [3]. In the Agache et al. [1] study the prevalence of uveitis in Romanian patients with AS was 14%. In the present study ocular involvement was %14.7. RAS in the vessel walls plays a crucial role in the endothelial control of vascular tonus and contributesto the inflammatory process and as known ACE is a regulatory component of the RAS, this may be related to ocular involvement in AS.

The frequency of *ACE* D allele in normal Caucasians is 50%–58%, but 35%–39% in normal Chinese [17-19]. In the present study D allele frequency in control group was 36.07% and I allele frequency was 63.93%. AS usually affects the younger age group and males. Accordance with this conclusion age mean of patients was 38.64±9.21 and female to male ratio was 0.9:1.

The exact pathogenesis of AS remains poorly understood but genetic factors play a key role in its development, accounting for more than 90% of the overall susceptibility to AS. Very little is known about the genetic control of disease severity in AS. Hamersma et al. used variancecomponents modeling to determine the genetic and environmental components in 384 patients and concluded that the disease severity is largely genetically determined and environmental factors play little role in determining the disease severity [20]. Based on large family and genome-wide association studies, the susceptibility to AS has been estimated to be 80%–90% genetically determined [21]. The average risk of developing AS in a first-degree relative of AS patients is about 8%, but only <1% in second-degree relatives. The risk in HLA–B27 positive first-degree relatives is about 12%, but <1% in HLA–B27-negative relatives [22,23].

There are limited number of studies about *ACE* I/D polymorphisms and AS in the literature. Shehab et al. [17] investigated the association between ACE I/D polymorphisms and inflammatory back pain (spondylarthropathies) secondary to AS, psoriatic arthritis, inflammatory bowel disease and undifferentiated spondylarthropathies in Kuwaiti Arabs. In their research *ACE* polymorphisms showed an overall significant difference between patients and controls. When the ID and II genotype frequency was combined and compared with that for DD genotype among patient and control groups, a considerably higher incidence was detected for ID and II genotypes than the DD genotype in spondylarthropathy patients compared to that in the controls (p=0.036).

Besides *ACE*, different gene polymorphisms were found associated with AS. Cosar et al. [24] reported that Familial Mediterranean Fever (FMF)-related Mediterranean fever (*MEFV*) variations were associated with AS, and these variations may contribute to the pathogenesis of AS, especially in populations in which the prevalence of FMF is high. Diaz-Pena et al. [25] showed a significant association (p<10−3) with AS in two markers in two different genes (CCR4-not transcription complex subunit 3 (*CNOT3*) and leukocyte-associated immunoglobulin-like receptor 2 (*LAIR2*)). Im et al. [26] reported that haplotypes of mannose-binding lectin (*MBL2*) genetic polymorphisms were found to be associated with AS . Huang et al. [27] suggest that the binding of programmed cell death 1 (*PD-1*) G-536A, *PD-L1* A8923C and *PD-L2* C47103T polymorphisms were associated with the presence of AS.

There are also several studies in Turkey in which *ACE* I/D polymorphisms were investigated. In some previous studies in dilated cardiomyopathy patients in the Turkey, Bayram et al. [5] did not found any difference in terms of the distribution of *ACE* I/D polymorphism genotypes between patients and controls. In a study in Turkish patients with insulin resistance, Akin et al. [28] did not also find any significant difference between patients and controls according to *ACE* I/D polymorphism genotypes. Whereas Bayram et al. [29] reported that DD homozygosis were found to be significantly higher in osteoarthritis than controls (DD) and D allele frequency and DD homozygosis were found to be significantly higher in polycystic ovary syndrome patients than controls in Turkish population. *ACE* I/D polymorphisms are also implicated in the pathogenesis of a variety of cardiovascular disorders and it has been recognized as a top candidate gene for cardiovascular research. A large number of studies have shown a positive association between the DD genotype and an increased risk of myocardial infarction [30]. Turgut et al. [31] was found statistically differences for *ACE* I/D polymorphism between BD patients and healthy controls (p=0.044). Ozturk et al. [32] evaluated 90 patients with BD and 30 control group for their *ACE* genotype and they reported that possession of either the D or the I allele does not have an impact on the development of BD. Additionally Dursun et al. [33] evaluated 73 patients with BD and 90 control for their *ACE* genotype and they reported that the *ACE* polymorphism does not play a role in the pathogenesis of BD. But in these studies, study groups were not large and ıt would not be enough to detect the difference.

Most recently, two new genetic loci have been shown to be associated with AS besides *HLA-B27.2*. These are interleukin 23 (*IL23*) receptor which is involved in the Thelper cell 17-pathway of immune responses and the other is endoplasmic reticulum associated protein (*ERAP1*), an enzyme which is relevant for the processing of peptides in the cytoplasm [34,35]. A meta-analysis showed that the rs27044, rs17482078, rs10050860, rs30187, and rs2287987 polymorphisms of endoplasmic *ERAP1*, were associated with the development of AS in Europeans [36].

Several studies have demonstrated that different genes play an important role in the etiology of AS. Due to the limited research about *ACE* and AS, present study provides an important contribution to the literature.

Our results suggest that, possession of D allele of the *ACE* polymorphism may constitute a risk for developing AS. Further work is required to confirm these findings in different study groups.
